# Lung Ultrasound in Predicting Outcomes in Patients with COVID-19 Treated with Extracorporeal Membrane Oxygenation

**DOI:** 10.3390/v15091796

**Published:** 2023-08-24

**Authors:** Valentin Sebastian Schäfer, Florian Recker, Edgar Kretschmer, Christian Putensen, Stefan Felix Ehrentraut, Christian Staerk, Tobias Fleckenstein, Andreas Mayr, Armin Seibel, Jens-Christian Schewe, Simon Michael Petzinna

**Affiliations:** 1 Department of Internal Medicine III, Oncology, Hematology, Rheumatology and Clinical Immunology, University Hospital of Bonn, 53113 Bonn, Germany; valentin.schaefer@ukbonn.de (V.S.S.); edgar.kretschmer@ukbonn.de (E.K.); 2 Department of Obstetrics and Gynecology, University Hospital of Bonn, 53113 Bonn, Germany; florian.recker@ukbonn.de; 3 Department of Anaesthesiology and Intensive Care Medicine, University Hospital of Bonn, 53113 Bonn, Germany; christian.putensen@ukbonn.de (C.P.); stefan.ehrentraut@ukbonn.de (S.F.E.); jens-christian.schewe@med.uni-rostock.de (J.-C.S.); 4 Institute for Medical Biometry, Informatics and Epidemiology, Medical Faculty, University of Bonn, 53113 Bonn, Germany; christian.staerk@ukbonn.de (C.S.); fleckenstein@imbie.meb.uni-bonn.de (T.F.); andreas.mayr@ukbonn.de (A.M.); 5 Department of Intensive Care Medicine, DRK Hospital Kirchen, 57548 Kirchen, Germany; arminseibel1@me.com; 6 Department of Anaesthesiology Intensive Care Medicine and Pain Therapy, University Medical Centre Rostock, 18057 Rostock, Germany

**Keywords:** coronavirus disease 2019 (COVID-19), severe acute respiratory syndrome coronavirus 2 (SARS-CoV-2), extracorporeal membrane oxygenation, lung ultrasound, acute respiratory distress syndrome, ultrasound, handheld ultrasound device

## Abstract

Pulmonary involvement due to SARS-CoV-2 infection can lead to acute respiratory distress syndrome in patients with COVID-19. Consequently, pulmonary imaging is crucial for management of COVID-19. This study aimed to evaluate the prognostic value of lung ultrasound (LUS) with a handheld ultrasound device (HHUD) in patients with COVID-19 treated with extracorporeal membrane oxygenation (ECMO). Therefore, patients underwent LUS with a HHUD every two days until they were either discharged from the intensive care unit or died. The study was conducted at the University Hospital of Bonn’s anesthesiological intensive care ward from December 2020 to August 2021. A total of 33 patients (median [IQR]: 56.0 [53–60.5] years) were included. A high LUS score was associated with a decreased P/F ratio (repeated measures correlation [rmcorr]: −0.26; 95% CI: −0.34, −0.15; *p* < 0.001), increased extravascular lung water, defined as fluid accumulation in the pulmonary interstitium and alveoli (rmcorr: 0.11; 95% CI: 0.01, 0.20; *p* = 0.030), deteriorated electrolyte status (base excess: rmcorr: 0.14; 95% CI: 0.05, 0.24; *p* = 0.004; pH: rmcorr: 0.12; 95% CI: 0.03, 0.21; *p* = 0.001), and decreased pulmonary compliance (rmcorr: −0.10; 95% CI: −0.20, −0.01; *p* = 0.034). The maximum LUS score was lower in survivors (median difference [md]: −0.35; 95% CI: −0.55, −0.06; *p* = 0.006). A cutoff value for non-survival was calculated at a LUS score of 2.63. At the time of maximum LUS score, P/F ratio (md: 1.97; 95% CI: 1.12, 2.76; *p* < 0.001) and pulmonary compliance (md: 18.67; 95% CI: 3.33, 37.15; *p* = 0.018) were higher in surviving patients. In conclusion, LUS with a HHUD enables continuous evaluation of cardiopulmonary function in COVID-19 patients receiving ECMO support therapy and provides prognostic value in determining the patients’ likelihood of survival.

## 1. Background

Coronavirus disease 2019 (COVID-19), caused by severe acute respiratory syndrome coronavirus 2 (SARS-CoV-2), is a parenchymal, endothelial disease with substantial lung involvement. This often manifests as a bilateral and diffuse interstitial pneumonia with asymmetric lesions and uneven distribution. While real-time polymerase chain reaction (RT-PCR) is frequently used to confirm the diagnosis [1], it does not provide diagnostic or prognostic value for evaluating disease severity and prognosis [2]. Therefore, clinical imaging has increasingly gained relevance in the assessment of COVID-19. The chest computed tomography (CT) scan, in particular, has proven to be a vital diagnostic tool. Studies have demonstrated its superior sensitivity over RT-PCR for diagnosing COVID-19. Early ground-glass opacities originating in subpleural zones, interlobular septal thickening, pleural thickening, progressive extension, and consolidation have all been observed in CT studies [3,4,5].

Despite these advantages, the use of CT thoracic imaging in pulmonary diagnostics presents certain limitations, including radiation exposure, restricted availability, high cost, and the need for intrahospital transfer. As a result, lung ultrasound (LUS) has emerged as a feasible noninvasive bedside alternative to evaluate various lung diseases such as pulmonary edema, pneumonia, interstitial lung disease, and associated acute respiratory distress syndrome (ARDS) [6,7,8,9]. Thus, LUS can be performed as real-time point-of-care ultrasound (POCUS) with a handheld ultrasound device (HHUD) at bedside. Different scoring systems scanning pre-determined pulmonary zones have been proposed and evaluated for a standardized LUS, thereby composing the LUS score [10,11,12,13,14,15]. Several studies have indicated LUS equaling CT scan in detecting interstitial lung disease [9,16,17] and have revealed its predictive capacity for outcome in pulmonary diseases [18,19].

Given the subpleural and peripheral lung involvement in COVID-19 patients, LUS appears to be a particularly suitable diagnostic tool [20,21,22]. Thus, LUS reveals a typical pattern of diffuse interstitial lung syndrome, characterized by multiple or confluent bilateral B-lines. These predominantly represent vertical, non-specific artifacts originating in the visceral pleura, resulting from an increase in subpleural lung water [20,23,24,25]. Moreover, spared areas, thickening of the pleural line with pleural line irregularity, and subpleural and translobular consolidations with dynamic air bronchogram can be detected [23,24,26,27,28,29].

Multiple studies have explored the diagnostic value of LUS in patients with COVID-19, indicating that ultrasound imaging results align with results of CT findings [3,5,28,30]. A high sensitivity and negative predictive value for the diagnosis of COVID-19 could be shown [31,32], increasing with the severity of COVID-19 [5,33]. In this context, LUS has been shown as a predictor of the duration of mechanical ventilation, disease progression, treatment response, and prognosis [22,34,35,36,37]. However, only a few studies have explored the use of LUS in COVID-19 induced ARDS patients undergoing extracorporeal membrane oxygenation (ECMO) support therapy. While Møller-Sørensen et al. suggested that patients with a lower LUS score were more likely of being weaned from ECMO support therapy [38], Lazerri et al. evaluated LUS score as a predictor of death in intensive care unit (ICU) death [39].

Utilizing our expertise, we present an extensive sample of COVID-19 patients with ARDS who received ECMO support therapy and underwent regular LUS assessments at two-day intervals. Our aim was to assess the prognostic efficacy of LUS through HHUD in COVID-19 patients supported by ECMO within an ICU environment.

## 2. Materials and Methods

From December 2020 to August 2021, individuals diagnosed with COVID-19 and undergoing ECMO support therapy were recruited at the University Hospital Bonn. Eligibility criteria included a confirmed diagnosis of COVID-19, undergoing treatment with ECMO support in the ICU, age above 18 years, and free consensus to participate of the patient, next of kin, or legal representative. All those not fulfilling the inclusion criteria were excluded.

### 2.1. Ultrasound Examination

Within 24 h of initiating ECMO treatment, the first LUS examination was conducted. Throughout their ECMO support therapy, patients were consistently examined every second day until they were either discharged from the ICU or passed away. For the LUS examinations, we utilized the ButterflyiQ (Butterfly Network, Guilford, CT, USA) portable, pocket-sized ultrasound scanner probe. This probe operates at a frequency of 1–10 MHz, offers a scan depth range of 2–30 cm, and is equipped with a silicon chip. The chip features a 2-D array of 9000 capacitive micro-machined ultrasound transducers and is capable of emulating curved, linear, or phased transducers on-demand in M-mode, B-mode, or color Doppler. The examinations were conducted using a lung preset for each session. This preset is optimized for displaying and sliding of the pleural surface, and for visualizing A-line and B-lines throughout the 15 cm field. The preset transitions from a curvilinear array to a linear one when the depth is reduced to 7 cm or less, further enhancing near-field lung sliding dynamics and pleural artifacts. The investigator was permitted to adjust image depth and focus point as necessary. The depth was adjusted so that 5–6 cm of the lung tissue was visualized. Before the initiation of the study, the lung preset and its adaptation were rigorously tested on individuals to ensure satisfactory imaging quality by a single expert sonographer with the highest degree for lung ultrasound (EFSUM/DEGUM level III). Examination was performed intercostal with cineloops of at least 5 s to capture enough breathing cycles to assess the whole area. If the initial 5 s interval did not provide adequate information, we extended the recording interval to capture more comprehensive data. In total, 9052 loops were generated and analyzed. The LUS examination was performed by the investigator after training under guidance of a single expert sonographer with the highest degree for lung ultrasound (EFSUM/DEGUM level III) until the quality of the imaging data was approved. Throughout the duration of the study, the investigator received feedback on the quality of the generated imaging data by the specialist.

### 2.2. Scoring System

**Figure 1:**
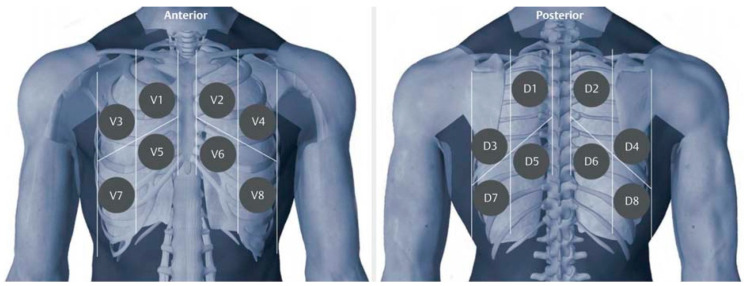
Scanning zones for intercostal lung ultrasound. Scanning zones for imaging data generation to assess ultrasound changes.

Each lung lobe was divided in four zones in the front and in the back (Figure 1). The scoring was performed according to the established system by Soldati et al. [40]. Zero points: normal LUS, one point: presence of three or more B-lines or pleural irregularities, two points: confluence of B-lines or small subpleural lesions and three points: lung consolidation (Table S1). The generated image data files were subsequently analyzed offline by a fully blinded analyst (EFSUMB Level III). Observations for each zone were added to a total score for each patient. If multiple lesions were detected in a zone, the largest lesion was scored. The maximum score achievable in each zone was three, culminating in a total possible score of 48 per patient. To account for observation bias due to sometimes-incomplete coverage of all lung zones (252 out of 582 examinations), our final LUS score was calculated by dividing the total points obtained by the number of zones examined. In addition, the LUS score Max, the highest LUS score attained by the patient on ECMO support therapy, was assessed.

### 2.3. Clinical Assessement

Various additional clinical and laboratory parameters were assessed. Simplified Acute Physiology Score II (SAPS II) (without Glascow Coma Scale calculation) and Therapeutic Intervention Scoring System 10 (TISS 10) were determined on a regular daily basis. Cardiopulmonary situation such as hemodynamic status (extravascular lung water), P/F ratio, arterial oxygenation, catecholamine circulatory support, and pulmonary compliance were recorded. Regular measurements of laboratory parameters including pH value, base excess, procalcitonin, lactate, and ferritin were taken and monitored. ECMO settings were recorded for each patient.

### 2.4. Statistical Analysis

Descriptive statistics of categorical variables are presented as absolute and relative frequencies, while for continuous variables medians and interquartile ranges are provided. To assess statistical differences between the groups of discharged (survival) and deceased (death) patients, Fisher’s exact tests were used for categorical variables and Mann–Whitney U tests were used for continuous variables. Effect sizes are presented as odds ratios and median differences between the discharged and deceased groups with corresponding two-sided 95% confidence intervals, respectively. Descriptive classical Pearson correlations between LUS and several parameters (clinical and laboratory) were calculated considering all examinations with available data; furthermore, to account for dependencies between multiple measurements on the same patients, repeated measures correlations were computed together with two-sided 95% confidence intervals and *p*-values [41]. A cutoff value for survival based on the maximum LUS score (LUS score Max) was calculated using the Youden Index. The occurrence of different pathologies was assessed by computing mean occurrence scores for each patient and pathology, averaged over all available examinations and regions; for each pathology, mean occurrence scores were compared between the discharged and deceased groups using Mann–Whitney U tests.

### 2.5. Ethical Approval

Aligned with the principles of the Declaration of Helsinki, this study received ethical clearance from the University Hospital Bonn’s ethics committee in Germany (reference number: 164/20), in July 2020. According to the ethics approval and the patients’ condition, written informed consent was obtained either from the patient, next of kin, or their caretakers/legal representative prior to inclusion.

## 3. Results

### 3.1. Patients’ Characteristics

The study included 33 patients (age: median [IQR], 56.0 [53–60.5] years; 17 [58%] female) (Table S2). Overall, mortality was high (70%). Survival was slightly higher in women (35%) than in men (25%) (*p* = 0.71). The median observed survival was 29 days (IQR, 20.75–38.5). The median duration of ECMO support therapy was 21 days (IQR, 16–28). On average (median), 7.5 areas (IQR, 6–8) were analyzed per examination resulting in a median of 13.5 scoring points (IQR, 12–18). The median LUS score was 2.0 (IQR, 1.63–2.38).

### 3.2. Pathologies in Lung Ultrasound

**Figure 2:**
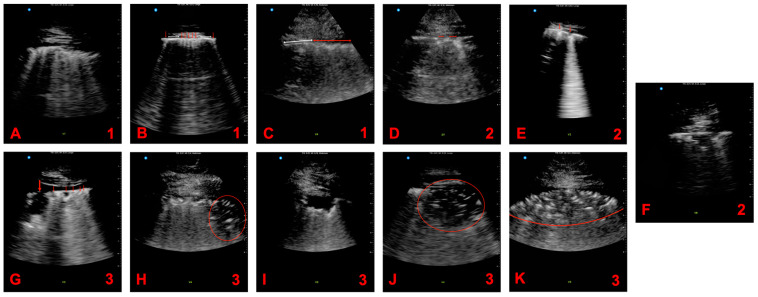
Example images of pathologies detected with lung ultrasound. (**A**): Thickened pleura with multi-focal B-lines. (**B**): Multi B-lines (red arrows) surrounded by normal regions (white lines). (**C**): Distinct thickened pleura (red line) with sharp border to normal pleura (white line). (**D**): Pleural line interruptions due to minor subpleural consolidations (red lines). (**E**): Isolated small subpleural consolidations (red arrows). (**F**): Demarcated subpleural consolidation without airbronchogram. (**G**): Large consolidation in the right ventral segment 3 (large red arrow). Nearby, smaller consolidations (small red arrows) are seen below a well-defined smooth pleural line. (**H**): Widespread lung surface infiltration. The pleural line, thickened and fragmented over the entire sector, with numerous subpleural consolidations merging caudally into a larger consolidation (red circle) with residual air (static airbronchogram). (**I**): Pronounced consolidation without an airbronchogram, delineated sharply. The sharp boundary and absence of residual air artifacts indicates a region of infection-destroyed parenchyma rather than a subpleural aeration defect. (**J**): Typical pneumonic infiltrate with disseminated airbronchogram and diminished lung sliding, signifying reduced lung surface elasticity in the affected area. (**K**): Extensive infiltrate with a static airbronchogram observed in motion, obscuring the pleural line. The infiltrate’s boundary (red line) adjoins the normally aerated lung tissue; assessed scoring in upper right corner. All pathologies, barring G, were observed in deceased patients.

The most frequently observed pathologies in our study population were pulmonary consolidation with static airbronchogram (death: 18.0% vs. discharge: 6.0%, *p* = 0.001), small subpleural consolidation (death: 20% vs. discharge: 21%, *p* = 0.400), and irregular pleural lines (death: 15% vs. discharge: 19%, *p* = 0.092). Table S3 provides detailed information regarding the frequency of the detected pathologies in relation to patient outcomes, while Figure 2 illustrates example images of these pathologies.

### 3.3. LUS Score

**Figure 3:**
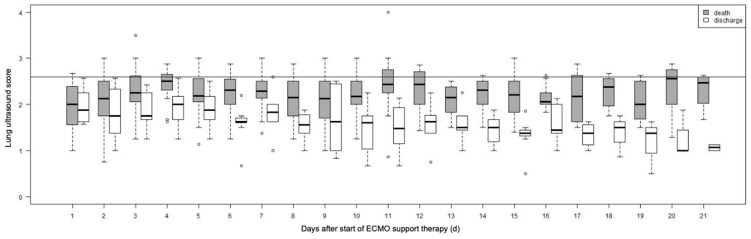
Lung ultrasound score in deceased and discharged patients. Progression of lung ultrasound score observed after initiation of ECMO treatment in deceased (death) and surviving (discharge) patients. Abbrv. ECMO: extracorporeal membrane oxygenation.

Figure 3 depicts the evolution of the LUS score throughout the disease. We observed a significant negative correlation between the P/F ratio and the LUS score (repeated measures correlation [rmcorr]: −0.26; 95% CI: −0.34, −0.15; *p* < 0.001). Similarly, the LUS score was significantly correlated with hemodynamic status (rmcorr: 0.11; 95% CI: 0.01, 0.20; *p* = 0.031), base excess (BE) (rmcorr: 0.14; 95% CI: 0.05, 0.24; *p* = 0.004) as well as pH (rmcorr: 0.12; 95% CI: 0.03, 0.21; *p* = 0.001). A significant link was identified between reduced pulmonary compliance and LUS score (rmcorr: −0.10; 95% CI: −0.20, −0.01; *p* = 0.034) and we also found a negative correlation between catecholamine-based circulatory support and LUS score (rmcorr: −0.10; 95% CI: −0.19, −0.004; *p* = 0.040) (Table 1).

A high lung ultrasound score was associated with a decreased P/F ratio, an increased extravascular lung water, a deteriorated electrolyte status, increased catecholamine requirement, and decreased pulmonary compliance. Simplified Acute Physiology Score II, Therapeutic Intervention Scoring System 10, procalcitonin, lactate, and ferritin did not correlate with lung ultrasound score (data not shown). Abbr. LUS: lung ultrasound, rmcorr: repeated measures correlation

### 3.4. LUS Score Max

**Figure 4:**
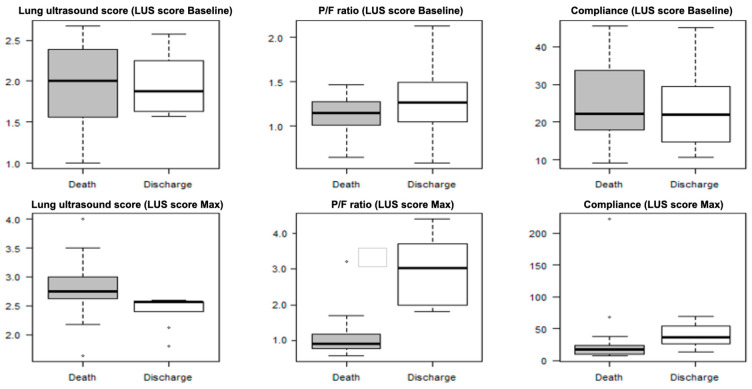
Assessment of baseline and maximum lung ultrasound score. LUS score Max was lower in surviving (discharge) patients compared to deceased (death) patients. Furthermore, at the time where the maximum lung ultrasound score was obtained, the P/F ratio and the pulmonary compliance were larger in surviving (discharge) patients. Abbr.: LUS: lung ultrasound score, LUS score Baseline: lung ultrasound score at initiation of ECMO support therapy, LUS score Max: maximum lung ultrasound score assessed, ECMO: extracorporeal membrane oxygenation.

The mean period from reaching the maximum LUS score (LUS score Max) and the end of follow-up (death or discharge) was 15.9 days (± 11.4). LUS score Max during ECMO therapy was significantly lower in discharged patients compared to deceased patients (median difference [md]: −0.35; 95% CI: −0.55, −0.06; *p* = 0.005). The cutoff value for survival based on LUS score Max was 2.63 with a sensitivity of 0.64 and a specificity of 1. At the time where the LUS score was maximal, the P/F ratio (md: 1.97; 95% CI: 1.12, 2.76; *p* < 0.001) and the pulmonary compliance (md: 18.67; 95% CI: 3.33, 37.15; *p* = 0.018) were larger in discharged patients compared to deceased patients (Figure 4). At baseline (initiation of ECMO therapy), there were no significant differences in LUS score, P/F ratio, or lung compliance between patients who died and those who could be discharged.

## 4. Discussion

In our investigation, we identified a noteworthy inverse correlation between the LUS score and the P/F ratio in patients with COVID-19 treated with extracorporeal membrane oxygenation. This finding is consistent with prior research by Dargent et al., which observed a similar link in COVID-19 patients with ARDS without ECMO support therapy. Additionally, their work indicated connections to disease progression and the likelihood of ventilator-associated pneumonia [22]. However, the correlation between the LUS score and the P/F ratio observed in our study was only moderate. This might be partially due to the limited ability of LUS to assess central lung sections. While LUS is effective in assessing inflammatory processes at the air-lung tissue interface, its technical limitations impede its ability to visualize deep lung lesions not extending to the pleural surface. Nonetheless, our findings are significant, particularly given the sample size. The LUS score appears to enable quantitative estimation of the lung surface area affected and available for oxygenation, thereby allowing monitoring of the pathologic course of COVID-19 and the impact of therapeutic strategies. However, due to the limiting imaging aspects, LUS alone may not provide definitive evidence of an oxygenation disorder. Further studies with larger sample sizes may help to strengthen the correlation between the aforementioned aspects.

Considering the ECMO support therapy, the absence of a correlation between LUS score and arterial oxygenation in our study appears plausible. The correlation between LUS score and an increasing base excess could reflect a decline of circulatory capability and a consecutive metabolic acidosis. The association between LUS score and hemodynamic status, represented by the accumulation of extravascular lung water, further underscores the value of LUS as a tool for the assessment of cardiopulmonary function deterioration. In the context of deteriorated pulmonary function, correlation was also found between pulmonary compliance and LUS score. This is of particular importance, as no correlation could be detected in previous studies involving COVID-19 patients with ARDS [22]. However, this appears reasonable in consideration of the stage of disease of our study population. The more affected the involved lung tissue, the higher the LUS and the higher the stiffness of the lung.

Within our study, ultrasound manifestations of COVID-19, an inherently heterogeneous disease with highly variable courses, appear to differ to some extent compared to previously published studies. Thus, in our study, (multifocal) B-lines could only rarely be shown. These B-lines are frequent findings especially in patients in the early stages of the disease [26]. This (along with the fact that B-lines decreased further during the course of the disease in the deceased patients) suggests that B-lines are associated with more moderate clinical courses, which is already reflected to some extent in the LUS score by the higher scoring of other findings.

Multiple studies have underscored the importance of subpleural manifestations in LUS in COVID-19 patients [39,42,43,44]. While these can appear in a variety of diseases, they, in combination with B-lines, represent typical patterns in ARDS patients [26]. It has been demonstrated that these (sub-)pleural manifestations can be seen as a sign of progression of interstitial manifestation in the context of COVID-19 disease advancement. In line with these findings, our study shows a significantly more diseased patient population than previous studies. Owing to the variable course of COVID-19, LUS results across different lung zones may diverge if they are at different stages of disease.

A key element of our study was the exploration of the prognostic value of the LUS score in patients with COVID-19 receiving ECMO support therapy. Previous research has identified the LUS score as a predictor of ICU admission, extubation failure in elderly patients, and refractory disease on the ICU [11,22,45,46]. Moreover, it has been shown that handheld ultrasound devices offer a promising alternative to repeated X-ray examinations for monitoring peripheral lung diseases [47]. In our study, we found that the LUS score Max was highly predictive for the prognosis of COVID-19 patients on ECMO support therapy. We established a link between the LUS score Max, P/F ratio, and lung compliance and further discovered that patients with a high LUS score Max were significantly less likely to survive. For the first time, we were able to define a prognostic cutoff value for the LUS score Max for non-survival in patients with COVID-19 treated with ECMO support therapy. No patient with a LUS score higher than 2.6 survived the treatment. The prognostic utility of the LUS score Max is further enhanced considering the median time interval of 16 days between LUS score Max and death or discharge from ICU, providing physicians a timely and precise indicator to assess the prognostic benefit in proceeding further treatment.

In our study, the use of LUS in COVID-19 patients with ECMO support proved not only to be easily applicable and administrable but also presented significant operational advantages. Thus, an examination took less than five minutes and allowed quick and repeatable real-time monitoring of disease progression without the need to move critically ill and difficult-to-transport patients, as is the case for CT scans. Moreover, the use of the HHUD mitigated the risk of contamination and infection of materials and staff, minimizing the burden on the critically ill patient and preserving resources compared to intrahospital transport. Despite the compromised image quality compared to conventional high-end ultrasound machines, the HHUD provided valid results.

A brief intensive training enabled a previously inexperienced examiner to produce high-quality, reproducible results with prognostic significance. However, considering prior studies like that of Šustić A et al. [48], which highlighted inconsistent interrater reliability in LUS for COVID-19, our study might not be exempt from these discrepancies. Even with rigorous training and standardization in our study, the inherent challenges of LUS interpretation for COVID-19 cannot be fully dismissed. Enhancing and standardizing training further could potentially elevate diagnostic quality and reproducibility [10].

While our study offers valuable insights into the prognostic utility of LUS in critically ill COVID-19 patients receiving ECMO support therapy, it is crucial to acknowledge its limitations. Despite being the most extensive study to date using HHUD on COVID-19 patients under ECMO support therapy, we worked with a relatively small patient population exhibiting a high mortality rate. This high mortality can introduce biases, notably the survivorship bias, which may affect the interpretation of our results. Given the limited sample size, direct generalizability of our findings to broader populations might be challenging. Further multicenter studies with a larger sample size are needed to obtain additional data on the prognostic value of LUS in COVID-19 patients with ECMO support therapy and to validate the LUS score Max cutoff value. The LUS score Max cutoff’s inherent limitation is that it is only prospectively informative if the cutoff is surpassed. Moreover, the reliance on a delayed-time approach as opposed to real-time assessments, as the observer analyzed the LUS from recorded data rather than live observations, may influence the results of the LUS assessment. While this approach allowed for calm analysis and the possibility of revisitation, it might have compromised the immediacy and contextual accuracy. Future studies should consider incorporating real-time evaluations to achieve a more contextually precise interpretation of the LUS readings. Other limitations are related to the limitations of LUS in general. Evaluation of the pleura and subpleural areas in obese patients is limited due to reduced tissue penetration. A different number of data were obtained in the prone and supine positions as a result of varying positioning in the hospital bed. Thus, examination of the anterior and posterior zones was not always possible. Undoubtedly, the supine position and ECMO intervention present considerable obstacles when striving for thorough LUS evaluations. These factors can impact the visibility and lucidity of distinct lung regions, possibly resulting in incomplete or inconclusive outcomes. The potential diagnostic advantage of observing and analyzing 16 zones in 5 s loops, as conducted in our study, remains unclear when compared to other proposed scoring systems. More research is required to determine the prognostic value in relation to the number of regions studied. An integrated approach combining clinical and LUS findings has not been conducted and could be the subject of further studies.

## 5. Conclusions

To the best of our knowledge, our study represents the largest prospective study conducted in patients with COVID-19 induced ARDS requiring ECMO support therapy. We demonstrated that frequent bedside real-time assessment using LUS and HHUD provides indications of oxygenation impairment, although it may not conclusively diagnose such disorders on its own. Moreover, we demonstrated the high prognostic utility of the LUS score concerning patient outcomes, identifying a cutoff value for survival prediction. Thus, easily available, resource-efficient and rapid examination with LUS offers a valid alternative to existing imaging methods for the assessment and prognosis of the course of COVID-19 associated ARDS within ECMO support therapy and can therefore be incorporated for treatment management in the ICU.

## Figures and Tables

**Table 1:** Clinical/ laboratory assessment and correlations with lung ultrasound score.

Parameter	n	Classical Correlation with LUS Score	Repeated Measures Correlation [rmcorr] with LUS Score	95% CI Low [rmcorr]	95% CI High [rmcorr]	*p* Value [rmcorr]
**Hemodynamic status (Extravascular lung water)**	444	0.36	0.11	0.01	0.20	0.031
**P/F ratio**	425	−0.40	−0.26	−0.34	−0.15	<0.001
**pH value**	470	0.19	0.12	0.03	0.21	0.001
**Pulmonary compliance**	449	−0.21	−0.10	−0.20	−0.01	0.034
**Catecholamine circulatory support**	478	0.14	−0.10	−0.19	−0.004	0.040
**Base excess**	429	0.12	0.14	0.05	0.24	0.004

## Data Availability

Data are available upon reasonable request from the corresponding author.

## Supplementary Materials

The following supporting information can be downloaded at: https://www.mdpi.com/article/10.3390/v15091796/s1, Table S1: Lung ultrasound scoring according to Soldati et al. [40], Abbrev.: LUS: lung ultrasound score; Table S2: Patient characteristics and outcome. Assessed patient characteristics, comparing patients deceased and discharged from intensive care unit. Estimated effect sizes are given as Odds Ratios for categorical variables and as estimated differences for continuous variables together with 95% confidence intervals, Abbrev.: (Δ): Estimated difference, (OR): Odds Ratio; Table S3: Assessement of detected pathologies. Occurrence of the detected pathologies in relation to the outcome of the patients.
