# Supplementary material for: Lung Ultrasound in Predicting Outcomes in Patients with COVID-19 Treated with Extracorporeal Membrane Oxygenation
Source: Viruses. 2023 Aug 24;15(9):1796. doi: 10.3390/v15091796 (PMC10535976; doi:10.3390/v15091796)
Supplement: Supplementary file 1 [file viruses-15-01796-s001.zip › viruses-2543456-supplementary.pdf]

**Pathologies**	**LUS**
No pathologies	0
Multi-focal B-lines (>2 ICS)	1
Focally fixed B-lines (without respiratory echodynamics)	1
Thickened pleural line	1
Uneven pleural line (retractions, irregular line)	1
Confluent B-lines	2
Light beam	2
Interruptions in the pleural line	2
Subpleural lesions (small)	2
Subpleural consolidations (with dynamic airbronchogram)	2
Subpleural consolidations (with static airbronchogram)	3
Subpleural consolidations (without airbronchogram)	3
Subpleural consolidations (with reduced perfusion)	3

Table S1: Lung ultrasound scoring

Lung ultrasound scoring according to Soldati et al. [40], Abbrev.: LUS: lung ultrasound score.

Parameter	total (n=33)	discharge (n=10)	death (n=23)	(Δ) / (OR)	CI low	CI high	p-value
**Females [N; %]**	17 (53%), 1 Missing	6 (60%)	11 (50%), 1 Missing	OR = 0.68	0.11	3.82	0.71
**Days in ICU [Median; IQR]**	29 (20.75 – 38.5)	22 (18.5 – 33.75)	30 (22,8-35,5)	Δ = 5	-5.00	13.00	0.37
**Prone/ supine position [N; %]**	19 (66%), 4 Missings	6 (67%), 1 Missing)	13 (65%), 3 Missings)	OR = 1.07	0.16	8.72	1
**Age (years) [Median; IQR]**	56 (53 – 60.5)	55 (48 60)	57 (54 – 61.5)	Δ = 5.00	-3.00	12.00	0.28
**BMI [Median; IQR]**	30.12 (28.37 – 35.92)	33.01 (29.57 – 36.75)	29.39 (27.76 – 35.21)	Δ = - 1.88	-7.38	1.52	0.15
**ECMO duration (days) [Median; IQR]**	21 (16 – 28)	18 (16.25 – 24.55)	23 (17 – 28.5)	Δ = 3.00	-5.00	10.00	0.79
**Immunmodulatory therapy [N; %]**	15 (47%), 1 Missing	5 (50%)	10 (45%), 1 Missing	OR = 0.84	0.14	4.84	1

**Total Score**<br><br>**[Median; IQR]**	13.5<br><br>(12 – 18)	15<br><br>(13 – 18)	13 (12 – 18)	Δ<br><br>=<br><br>1.00	-5.00	2.00	0.52
**Examined areas**<br><br>**[Median; IQR]**	7.5<br><br>(6 - 8)	8<br><br>(7 – 8)	7 (6 – 8)	Δ<br><br>=<br><br>0.00	-2.00	1.00	0.59
**LUS**<br><br>**[Median; IQR]**	2<br><br>(1.63 – 2.38)	1.88<br><br>(1.62 – 2.25)	2 (1.57 – 2.39)	Δ<br><br>=<br><br>0.04	-0.37	0.43	0.79

Table S2: Patient characteristics and outcome

Assessed patient characteristics, comparing patients deceased and discharged from intensive care unit. Estimated effect sizes are given as Odds Ratios for categorical variables and as estimated differences for continuous variables together with 95% confidence intervals, Abbrev.: (Δ): Estimated difference, (OR): Odds Ratio.

**Pathologies**	**Death mean**	**Discharge mean**	**p-value**
**No pathologie**	0.022	0.027	0.06
**Multi-focal B-lines (>2 ICS)**	0.015	0.016	0.58
**Focally fixed B-lines (without respiratory echodynamics)**	0.0003	0.000	0.37
**Thickened pleural line**	0.10	0.098	0.8
**Uneven pleural line (retractions. irregular line)**	0.15	0.19	0.09
**Confluent B-lines**	0.003	0.0008	0.98
**Light beam**	0.0015	0.001	0.93
**Interruptions in the pleural line**	0.050	0.02	0.15
**Subpleural lesions (small)**	0.196	0.21180	0.4
**Subpleural consolidations (with dynamic airbronchogram)**	0.000	0.0006	0.03
**Subpleural consolidations (with**	0.18	0.06	0.001

**static airbronchogram)**			
**Subpleural consolidations (without airbronchogram)**	0.012	0.010	0.77
**Subpleural consolidations (with reduced perfusion)**	0.000	0.002	0.93

Table S3: Assessement of detected pathologies

Occurrence of the detected pathologies in relation to the outcome of the patients.
